# A Report on the Health and Mortality of the City of Memphis

**Published:** 1853-08

**Authors:** 


*A Report on the Health and Mortality of the City of Memphis.* By CHAS. TODD QUINTARD, M. D.

Here we have one of those papers which every medical society should exact from its members. Dr. Quintard has embodied some statistics in this report, which must be of considerable local and general value. S. B. H.

